# Recombinant Costimulatory Fusion Proteins as Functional Immunomodulators Enhance Antitumor Activity in Murine B16F10 Melanoma

**DOI:** 10.3390/vaccines8020223

**Published:** 2020-05-14

**Authors:** Huaman Cai, Wenfang Wang, Zhibing Lin, Yan Zhang, Bing Wu, Yuhua Wan, Rongxiu Li

**Affiliations:** 1 State Key Laboratory of Microbial Metabolism, School of Life Sciences and Biotechnology, Shanghai Jiao Tong University, Shanghai 200240, China; lucycaihua@sjtu.edu.cn (H.C.); wenfangwang791408665@sjtu.edu.cn (W.W.); zhibinglin@sjtu.edu.cn (Z.L.); yan68069183@sjtu.edu.cn (Y.Z.); wubing1990@sjtu.edu.cn(B.W.); wanyuh2015@sjtu.edu.cn (Y.W.); 2 Engineering Research Center of Cell & Therapeutic Antibody, Ministry of Education, Shanghai 200240, China

**Keywords:** cancer immunotherapy, OX40L, 4-1BBL, immunomodulator, tumor microenvironment

## Abstract

Blocking inhibitory signaling and engaging stimulatory signaling have emerged as important therapeutic modalities for cancer immunotherapy. This study aimed to investigate immunomodulatory features of three recombinant costimulatory ligand proteins in a mouse model, which are extracellular domains of OX40-ligand (OX40L), 4-1BB-ligand (4-1BBL), or two domains in tandem, fused with the transmembrane domain of diphtheria toxin (DTT), named DTT-COS1, DTT-COS2, and DTT-COS12, respectively. In vitro study showed that DTT-COS1 and DTT-COS12 had immunological activity increasing the ratio of CD8/CD4 T cells. Treatments with DTT-COS1 and DTT-COS12 dramatically generated immune protection against the B16F10 tumor challenge in both prophylactic and therapeutic efficacy. Furthermore, regarding tumor microenvironment (TME) immunomodulation, DTT-COS1 treatment increased the proportion of CD4+ effector T cells (Teff) and decreased the expression of a suppressive cytokine. Meanwhile, DTT-COS12 reduced regulatory T cells (Treg) and improved the level of stimulatory cytokines. In addition, endogenous antibodies against OX40L/4-1BBL were generated, which may help with antitumor responses. Unexpectedly, DTT-COS2 lacked antitumor effects in vitro and in vivo. Importantly, serum analysis of liver-function associated factors and pro-inflammatory cytokines demonstrated that treatments were safe formulations in mice without signs of systemic toxicity. Remarkably, DTT-COS1 and DTT-COS12 are functional immunomodulators for mouse B16F10 melanoma, creating practical preclinical value in cancer immunotherapy.

## 1. Introduction

T-cell activation is modulated by a number of factors that depend on costimulatory and inhibitory signals, in addition to T cell receptor (TCR) signaling that specifically recognizes complexes of peptide and major histocompatibility complex (MHC) [1,2,3]. Costimulatory agonists have been activating and supporting antitumor immune response by combination with immune checkpoint inhibition and other tumor-associated agents [3]. OX40L, tumor necrosis factor (ligand) superfamily, member 4, is a costimulatory ligand for OX40 and is expressed mainly on antigen-presenting cells (APCs), including dendritic cells, B cells, and macrophages [3]. Supporting studies suggested that OX40L–OX40 pathway promoted immune responses during T cell activation by establishing T cell memory as well as the expansion and survival of activated T cell subsets [4,5,6,7]. There are several OX40-specific agonistic antibodies in clinical trials, such as MEDI6469, used as monotherapy (NCT02559024) and in combination with immune therapeutic agents or therapeutic monoclonal antibodies in subjects with selected advanced solid tumors or aggressive B-cell lymphomas (NCT02205333). 4-1BBL, tumor necrosis factor (ligand) superfamily, member 9, is a costimulatory ligand of 4-1BB and is expressed mainly on activated APCs, including B cells, macrophages and matured dendritic cells [1,2,3]. It is reported that 4-1BBL–4-1BB pathway delivers costimulatory signals to resting T cells and plays a role in sustaining T cell responses after CD28 co-stimulation [8,9,10,11,12]. Additionally, 4-1BB/4-1BBL signaling can augment T-helper 1 (Th1) and T-helper 2 (Th2) associated cytokine secretion by T cells and enhance cytotoxic T cell response both in vivo and ex vivo [13,14,15,16]. Agonistic 4-1BB-specific antibodies have some antitumor effect as a monotherapy or combination trial treatment, such as urelumab [3]. However, serious hepatotoxicity was observed [17,18], which caused the termination of the urelumab Phase II trial in patients with melanoma (NCT02420938). Importantly, it has hardly been reported that treatment with OX40/4-1BB agonistic antibodies protects mice against tumor challenge in preventive models.

In some studies, the combination of recombinant costimulatory ligand proteins (OX40L/4-1BBL) with tumor-associated antigens [19], virus-based vaccines [20], and tumor lysate vaccines [21] have been generating antitumor activities in some mouse models. We wondered how much different costimulatory ligand proteins contribute to tumor immunotherapy with no requirement of tumor-antigen identification or customized products. To address this issue, we designed three recombinant proteins, the murine extracellular domain of OX40L or 4-1BBL, and in tandem were fused to DTT herein DTT-COS1, DTT-COS2, and DTT-COS12, respectively. Surprisingly, treatments with DTT-COS1 and DTT-COS12 produced some immunological activities in vitro and generated immune protection against B16F10 tumor challenge for preventive and therapeutic efficacy. Importantly, the proteins modulated infiltrating regulator/effector function and the production of functional cytokines in TME, produced endogenous antibodies that helped antitumor responses, as well as circumvented systemic toxicity. In general, costimulatory fusion proteins we designed are functional, practical, and safe immunomodulators for antitumor immune treatment, promoting the application of more effective products for cancer prevention and therapy.

## 2. Materials and Methods

### 2.1. Cell Lines and Animals

The mouse melanoma cell line B16F10 was obtained from the Cell Bank, Chinese Academy of Sciences (Shanghai, China), cultured in complete Dulbecco’s modified Eagle’s medium (Gibco by Invitrogen, NY, USA) containing 10% fetal bovine serum (FBS, Gibco by Life Technologies, Grand Island, NY, USA) and penicillin/streptomycin (100 mg/mL) at 37 °C under a humidified atmosphere with 5% CO_2_.

Female C57BL/6 mice (6–8 weeks old) were purchased from SLAC Laboratory Animal Centre (Shanghai, China) and housed under pathogen-free conditions. All animal procedures were approved by the institutional animal care and use committee (IACUC) of Shanghai Jiao Tong University, with ethics approval number A2019091.

### 2.2. Construction of Expression Vectors Construction

The extracellular domains of mouse OX40L (residues 51-198), 4-1BBL (residues 104-309) was generated by NEST-PCR amplifying using mouse spleen cDNA, and DTT (residues 202-378) expression vector was from our laboratory. DTT-COS1, DTT-COS2, or DTT-COS12 were amplified by overlapping PCR, and digested with Nde I and Xho I, cloned into expression vector pET-28a.

### 2.3. Protein Expression and Purification

The DTT-COS1, DTT-COS2, or DTT-COS12 expression vectors were transformed into *E. coli* Rosetta-gami B (DE3) cells, respectively. Expression of the His_6_-tagged proteins was induced with 1 mM isopropyl-β-D-thiogalactoside (IPTG) when the *E. coli* culture reached OD_600_ = 0.6. After culturing for an additional 20 h, the cells were collected by centrifugation, resuspended in PBS, lysed by sonication, and the debris removed by centrifugation. Purification of the supernatant was applied to His Trap HP column. The DTT-COS1, DTT-COS2, or DTT-COS12 proteins were further purified through Superdex G75 chromatography. The level of endotoxin was lower than 0.1 EU/mL by chromogenic Limulus Amebocyte Lysate assay (GenScript, Piscataway, NJ, USA).

### 2.4. Costimulatory Fusion Proteins Treatments

C57BL/6 were treated with DTT, DTT-COS1, DTT-COS2, DTT-COS12 (50 μg\200 μL) or PBS in the presence of aluminum hydroxide Gel adjuvant (300 μg\200 μL; Invitrogen, Carlsbad, CA, USA) and CpG ODN 1826 (30 μg\200 μL; synthesis) three times subcutaneously (s.c.) at 2-week intervals [22]. Mice serum samples and weight data were collected from treated mice on the seventh day after each injection. All analyses of serum were from mice treated with DTT-COS1, DTT-COS2, DTT-COS12, DTT, or PBS on the seventh day after the third treatment.

### 2.5. Ex vivo Stimulation

For a naïve state, spleen cells were harvested from naïve C57BL/6 mice and prepared into a single-cell suspension. ACK Lysis Buffer was used to remove the red blood cells. Splenocytes were cultured in total RPMI 1640 medium (RPMI 1640 supplemented with 10% FBS, 100 U/mL penicillin/streptomycin), stimulated with 0.5 μg/mL anti-CD3 (Clone 145-2C11) as signal 1, an amount (10 μg/10^6^ cells) of DTT-COS1, DTT-COS2, DTT-COS12, or DTT control protein, as signal 2 and 20 U/mL interleukin-2 (IL-2) (Primegene) as signal 3 [23]. After 72 h stimulation, supernatants from cultured medium were collected and the cells were washed 3 times with PBS, stained with anti-CD3-PE (clone 145-2C11, BD Biosciences), anti-CD4-FITC (Clone GK1.5, BD Biosciences) and anti-CD8a-APC (Clone 53-6.7, BD Biosciences), and analyzed by flow cytometry.

For an immunized state, spleen cells were harvested from DTT-immunized C57BL/6 mice on the seventh day after the third injection and prepared into a single-cell suspension. An ammonium chloride-potassium (ACK) Lysis Buffer was used to remove the red blood cells. Splenocytes were cultured in total RPMI 1640 medium (RPMI 1640 supplemented with 10% FBS, 100 U/mL penicillin/streptomycin), stimulated with an amount (50 μg/10^6^ cells) of DTT-COS1, DTT-COS2, DTT-COS12, or DTT control protein, and 150 U/mL IL-2 (Primegene). After 72 h stimulation, supernatants from cultured medium were collected and the cells were washed 3 times with PBS, stained with anti-CD3-PerCP (clone 17A2, eBioscience), anti-CD4-FITC (Clone GK1.5, BD Biosciences) and anti-CD8a-APC (Clone 53-6.7, BD Biosciences), and analyzed by flow cytometry. Ten mice were used for each experiment under different conditions in total with ex vivo stimulation.

### 2.6. Preventive and Therapeutic Tumor Models

For the preventive tumor models, C57BL/6 mice were injected s.c. with 7.5 × 10^4^ B16F10 tumor cells, nine days after the third costimulatory fusion protein treatment. For the therapeutic tumor models, mice were s.c. challenged with 1 × 10^5^ B16F10 tumor cells, subsequent three-time treatments of fusion proteins at weekly intervals. Tumor size was measured every 2 to 3 days with a caliper, and tumor volume calculated using the formula (width^2^ × length × 0.5). The tumor size and survival were recorded until the tumor volume were reached 2000 mm^3^ and mice were sacrificed for ethical reasons [24].

### 2.7. Enzyme-Linked Immunosorbent Assay (ELISA) for Antibody Titers, Interferon-γ (IFN-γ), Interleukin-6 (IL-6) and Interleukin-8 (IL-8) Secretion

The serums were treated with magnetic beads coupled with DTT to remove antibodies against DTT, and then the antibody titer and absorbance (1:200 dilutions) after each treatment were detected by ELISA. Secondary antibodies used were goat anti-mouse IgG-HRP, or goat anti-mouse IgG1-HRP, or IgG2b-HRP, or IgG2c-HRP, or IgG3-HRP, or IgM-HRP (1:5000 dilutions, Shanghai Immune Biotech Co. Ltd., Shanghai, China), and 3, 3′, 5, 5′-tetramethylbenzidine (TMB, TIANGEN, Beijing, China) as the substrate agent. The absorbance at 450 nm was measured by EnSpire 2300 ELISA reader (PerkinElmer, Waltham, MA, USA). 

The amount of IFN-γ secretion in the supernatants diluted 2-fold from ex vivo stimulation above were measured by sandwich ELISA using the mouse IFN-γ DuoSet ELISA kits (R&D Systems) following the manufacturer’s instruction. All standards and samples were assayed in duplicate and data were analyzed in Microplate Reader with a 6-parameter fit for the standard curve.

To assess inflammatory reactions, IL-6 and IL-8 concentrations of serum from mice treated with DTT-COS1, DTT-COS2, DTT-COS12, or PBS were measured by Mouse IL-6 ELISA Kit (BOS-20268) and Mouse IL-6 ELISA Kit (BOS-46967) following the manufacturer’s instruction. The absorbance at 450 nm was measured by EnSpire 2300 ELISA reader (PerkinElmer, Waltham, MA, USA).

### 2.8. Treg/Teff Ratio and Cytokines Expression in TME

Preparation of tumor-infiltrating lymphocytes (TILs) was carried out using mouse tumor-infiltrating lymphocytes isolated tissue fluid kit (TBD), with subsequent staining using anti-CD4-FITC (Clone GK1.5, BD Biosciences), and anti-Foxp3- Alexa Fluor^®^ 647 (Clone MF-14, BioLegend) according to the flow cytometric staining manufacturer’s instructions, and analyzed by flow cytometry.

Tumor tissue RNA was isolated using TRIzol (Takara, Tokyo, Japan). 500 ng total RNA was reverse transcribed into cDNA using PrimeScript™ RT Master Mix kit (Takara, Tokyo, Japan) according to the manufacturer’s instructions. Real-time PCR was performed using TB Green™ Premix Ex Taq™ II (Tli RNaseH Plus) (Takara, Tokyo, Japan) to analyze mRNA expression of IL-2, IFN-γ, IL-4, transforming growth factor-β1 (TGF-β1), OX40 and 4-1BB in different treatments (PBS, DTT, DTT-COS1, and DTT-COS12), following the manufacturer’s instructions (Wcgene Biotech, Shanghai, China).

### 2.9. Serum Transfer Models

The serums were from C57BL/6 mice treated with DTT-COS1, DTT-COS2, DTT-COS12, or PBS three times and complement was inactivated by incubation for 30 min at 56 °C. Serum (300 µL) was administered by intraperitoneal (i.p.) injection 6 h before s.c. injection of 1 × 10^5^ B16F10 cells [25]. Tumor size was measured every 2 to 3 days with a caliper, and tumor volume calculated using the formula (width^2^ × length × 0.5). The tumor size and survival were recorded until the tumor volume were reached 2000 mm^3^ and mice were sacrificed for ethical reasons.

### 2.10. Quantification of the Liver Injury

To assess liver injury, alanine aminotransferase (ALT) and aspartate aminotransferase (AST) concentrations of serum from mice treated with DTT-COS1, DTT-COS2, DTT-COS12, or PBS (in the presence of aluminum hydroxide Gel adjuvant and CpG ODN 1826, as mentioned in 2.4.) were detected using the kit (Jiancheng Biologic Project Co., Nanjing, China) following the manufacturer’s instructions. The absorbance at 510 nm was measured by EnSpire 2300 ELISA reader (PerkinElmer, Waltham, MA, USA).

### 2.11. Histology and Immunohistochemistry

Tissues (liver, kidney, and lung), and tumor sections were fixed in 4% phosphate-buffered formalin for 24 h, paraffin-embedded tissues were serially sectioned, and were stained with Harris hematoxylin and eosin (Shanghai Skyho Biotech Co, Shanghai, China). For immunohistochemistry, the following antibodies were used: rabbit polyclonal to CD4 (SH0051, 1:100 dilution, skyhobio), rabbit polyclonal to CD8 (SH0052, 1:1000 dilution, skyhobio).

### 2.12. Statistical Analysis

GraphPad Prism 6.0 and Image J was used to analyze data and assess the statistical significance of comparisons between groups by an ordinary one-way ANOVA test or unpaired two-tailed Student’s test. *p* values < 0.05 were considered to be significant. Data were representative of two independent experiments. Comparison of Survival Curves were analyzed by the Kaplan–Meier method and *p* values were calculated using the Log-rank (Mantel–Cox) test.

## 3. Results

### 3.1. Design and Biological Activities of Costimulatory Fusion Proteins

**Figure 1:**
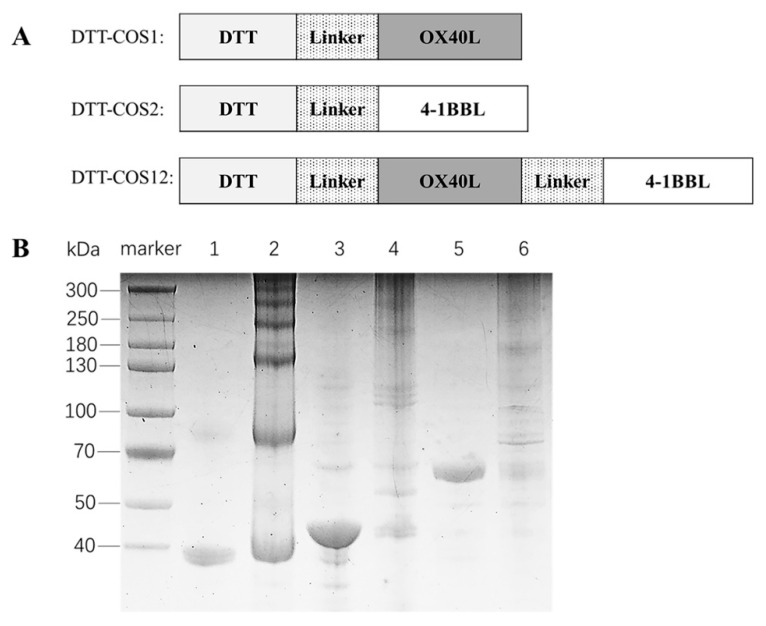
Design and expression of co-stimulatory fusion proteins. (**A**) A schematic diagram of DTT-COS1, DTT-COS2 and DTT-COS12. DTT are the transmembrane domain of diphtheria toxin and Linker sequence is GGGG. OX40L and 4-1BBL are the extracellular domains of mouse OX40L and 4-1BBL, respectively. (**B**) ExpressPlus^TM^ PAGE 4–12% gradient gel analysis of monomeric and oligomeric states of DTT-COS1, DTT-COS2 and DTT-COS12. To detect the monomeric state of proteins, the purified proteins were heated under reducing and denaturing conditions. Lane 1, 3 and 5 represent monomeric states of DTT-COS1, DTT-COS2 and DTT-COS12, respectively. To detect the oligomeric state of proteins, the purified proteins were mixed with native sample buffer without heating. Lane 2, 4 and 6 represent oligomeric state of DTT-COS1, DTT-COS2 and DTT-COS12, respectively.

We designed three fusion proteins and expressed soluble forms in *Escherichia coli*, which was infrequent in previous studies. The extracellular domains of OX40L, 4-1BBL, and two domains in tandem (4-1BBL, aa 104-309; OX40L, aa 51-198) were fused with DTT, named as DTT-COS1, DTT-COS2 and DTT-COS12 respectively (Figure 1A). Heated under reducing and denaturing conditions, DTT-COS1 (37.8 kDa), DTT-COS2 (44.1 kDa), and DTT-COS12 (61 kDa) were in monomeric states (Figure 1B). The native conformation of fusion proteins showed different oligomeric states (Figure 1B).

**Figure 2:**
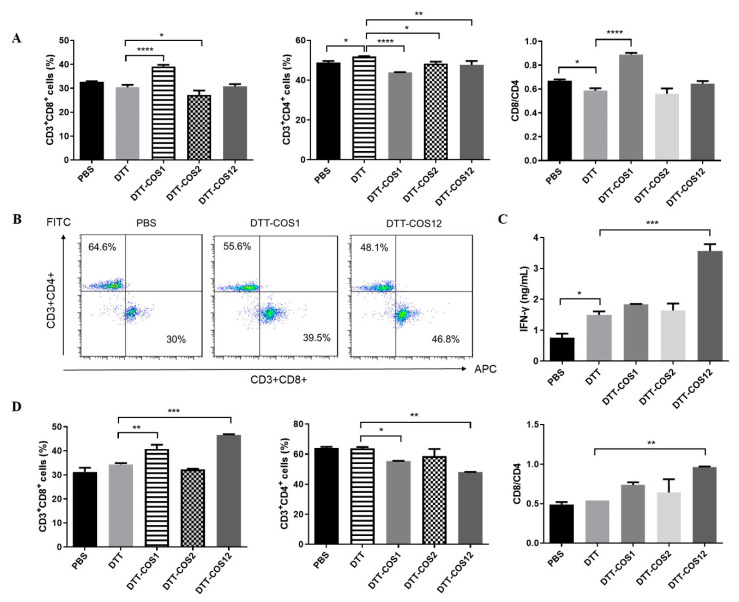
DTT-COS1 and DTT-COS12 increase the percentage of CD8+ T cells and expression of IFN-γ ex vivo. (**A**) In the presence of 0.5 μg/mL anti-CD3 and 20 U/mL IL-2, splenocytes from untreated C57BL/6 mice were stimulated 72 h with DTT-COS1, DTT-COS2, DTT-COS12, DTT control protein (10 μg/10^6^ cells) or PBS, respectively. The percentage of CD3+CD4+, CD3+CD8+ T cells and the ratio of CD8+/CD4+ in CD3+ T cells were analyzed by flow cytometry. (B-D) In the presence of 150 U/mL IL-2, splenocytes from DTT-immunized C57BL/6 mice were stimulated 72 h with DTT-COS1, DTT-COS2, DTT-COS12, DTT control protein (50 μg/10^6^ cells) or PBS, respectively. (**B**) Representative flow cytometry analysis of different T cell subpopulations in PBS, DTT-COS1, and DTT-COS12 treatments. (**C**) The secretion of IFN-γ in the supernatants were measured by ELISA. (**D**) The percentage of CD3+CD4+, CD3+CD8+ T cells and the ratio of CD8+/CD4+ in CD3+ T cells. Results are presented as means ± SD. Ordinary one-way ANOVA test was used to analyze the data compared to DTT group, **** *p* <0.0001; *** *p* <0.001; ** *p* < 0.01; * *p* < 0.05.

To assess biological activities of the costimulatory fusion proteins, spleen cells sorted from naïve or DTT-immunized C57BL/6 mice were stimulated for 3 days in the presence of soluble DTT-COS1, DTT-COS2, DTT-COS12 or DTT. For a naïve state, splenocytes were treated with signal 1 (0.5 μg/mL anti-CD3), signal 2 (10 μg/10^6^ cells costimulatory protein or DTT) and signal 3 (20 U/mL IL-2). After 72-h stimulation, the percentages of CD3+CD8+ T cells (~39%) had significantly increased in the DTT-COS1 group, compared to PBS (~32%) or DTT (~30%) (Figure 2A). The proportion of CD3+CD8+ T cells had slightly decreased by DTT-COS2 (~27%) stimulation, compared with DTT and DTT-COS12 (~31%) stimulation. In contrast, the percentages of CD3+CD4+ T cells (~44%) had decreased with DTT-COS1, compared to PBS (~49%) or DTT (~52%) (Figure 2A). The proportion of CD3+CD4+ T cells had slightly decreased by DTT-COS2 (~48%) or DTT-COS12 (~48%) stimulation, compared with DTT and PBS. What’s more, the proportion of CD8/CD4 was significantly increased in DTT-COS1 (~0.9) stimulation, compared to PBS (~0.66) or DTT (~0.58) (Figure 2A).

For an immunized state, splenocytes were stimulated with antigen DTT for a second time, signal 2 (50 μg/10^6^ cells costimulatory protein or DTT) and signal 3 (150 U/mL IL-2). Treatment with DTT-COS1 or DTT-COS12 had significantly increased the percentages of CD3+CD8+ (~42% and ~46.8%, respectively) compared to PBS (~30%), DTT (~34%), or DTT-COS2 (~32.5%) (Figure 2B,D). There was also a distinct decrease in the ratio of CD3+CD4+ T cells with DTT-COS1 and DTT-COS12 stimulation compared to PBS (Figure 2D). The proportion of CD8/CD4 was increased in DTT-COS1 (~0.74) or DTT-COS12 (~0.96) treatment, compared to PBS (~0.5) and DTT (~0.54) (Figure 2D). We further measured the secretion of IFN-γ in the cell culture supernatant by ELISA. In marked contrast to PBS (~0.7 ng/mL) or DTT (~1.5 ng/mL), DTT-COS12 treatment resulted in a striking increase in IFN-γ secretion, up to approximately 3.6 ng/mL (Figure 2C). A slight increase in DTT-COS1 (~1.8 ng/mL), DTT-COS2 (~1.6 ng/mL), as well as DTT (~1.5 ng/mL) treatment, compared to PBS (Figure 2C).

These results show that costimulatory fusion proteins, DTT-COS1 and DTT-COS12, have some immunological activities to increase the percentage of CD8+ T cells and the secretion of IFN-γ in vitro.

### 3.2. DTT-COS1 and DTT-COS12 Protect Mice against Tumor Challenge in the Prophylactic Model and Therapeutic Tumor Models In Vivo

**Figure 3:**
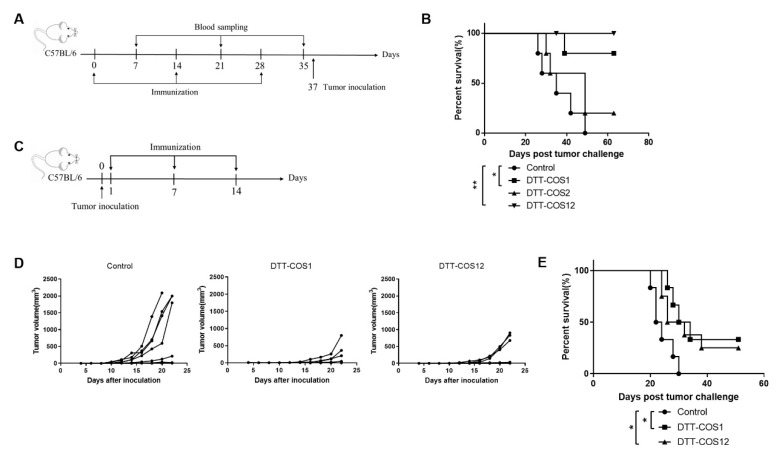
DTT-COS1 and DTT-COS12 protect mice against tumor challenge in the prophylactic and therapeutic model in vivo. (**A**,**B**) The C57BL/6 mice (*n* = 5) were treated with DTT-COS1, DTT-COS2, or DTT-COS12 (50 μg/200 μL) in the presence of aluminum hydroxide gel adjuvant (300 μg/200 μL) and CpG ODN 1826 (30 μg/200 μL) three times s.c. every 2 weeks. Serum were collected from treated mice on the seventh day after each injection. Nine days after the third treatment, the C57BL/6 mice were injected s.c. with 7.5 × 10^4^ B16F10 tumor cells. (**A**) Study design. (**B**) The Kaplan-Meier survival plot of mice subjected to control (PBS), DTT-COS1, DTT-COS2, or DTT-COS12. (C-E) The C57BL/6 mice were challenged s.c. with 1 × 10^5^ B16F10 tumor cells. One day after challenge, mice were treated with DTT (*n* = 6), DTT-COS1 (*n* = 6), or DTT-COS12 (*n* = 8) severally in the presence of aluminum adjuvant and CpG ODN 1826 three times s.c. at weekly intervals. (**C**) Study design. (**D**) The tumor growth curves for individual mouse treated control (DTT), DTT-COS1, and DTT-COS12. (**E**) The Kaplan-Meier survival plot of mice subjected to control (DTT), DTT-COS1, and DTT-COS12. The statistical significance was determined by Log-rank test. ** *p* < 0.01; * *p* < 0.05.

DTT-COS1 and DTT-COS12 significantly improved CD8+ T cells ratio and IFN-γ secretion in vitro, which indicated the proteins may elicit an antitumor immune response. To investigate the possibility, mice were pretreated three times with DTT-COS1, DTT-COS2, and DTT-COS12 respectively (50 μg/injection) 2 weeks apart, followed by B16F10 tumor challenge (Figure 3A). Consistent with the data in vitro, pretreatment with DTT-COS12 generated a long-lasting tumor-protective effect, as all mice in this group remained tumor-free for up to 60 days (Figure 3B). Similarly, pretreatment with DTT-COS1 also protected 80% of mice against the B16F10 tumor challenge (Figure 3B). In contrast, DTT-COS2 treatment did not have any effect on survival compared to control groups, as all mice developed tumors (Figure 3B). 

Based on the dramatically protective effect, we next assessed the therapeutic efficacy of DTT-COS1 and DTT-COS12. From the second day after s.c. inoculation of B16F10 tumor cells, C57BL/6 mice were treated three times with DTT, DTT-COS1, and DTT-COS12, respectively (50 μg/injection) 7 days apart (Figure 3C). Treatment with DTT-COS1 and DTT-COS12 resulted in potent inhibition of B16F10 tumor growth (Figure 3D). The survival of mice challenged with B16F10 tumor cells was significantly prolonged in the DTT-COS1 and DTT-COS12 treated groups when compared to the DTT group (Figure 3E). The median survival of DTT-COS1 and DTT-COS12 group were 32 and 29 days, respectively, while that of DTT control groups was 23 days (Figure 3E). Delaying tumor challenge by 6 or 9 days resulted in distinct retardation in tumor growth.

The results suggest a striking long-lasting tumor-protective effect on DTT-COS1 and DTT-COS12 treatments in vivo.

### 3.3. DTT-COS1 and DTT-COS12 Modulate the Proportion of Treg/Teff Cells and Expression of Cytokines in TME Following Therapeutic Models

**Figure 4:**
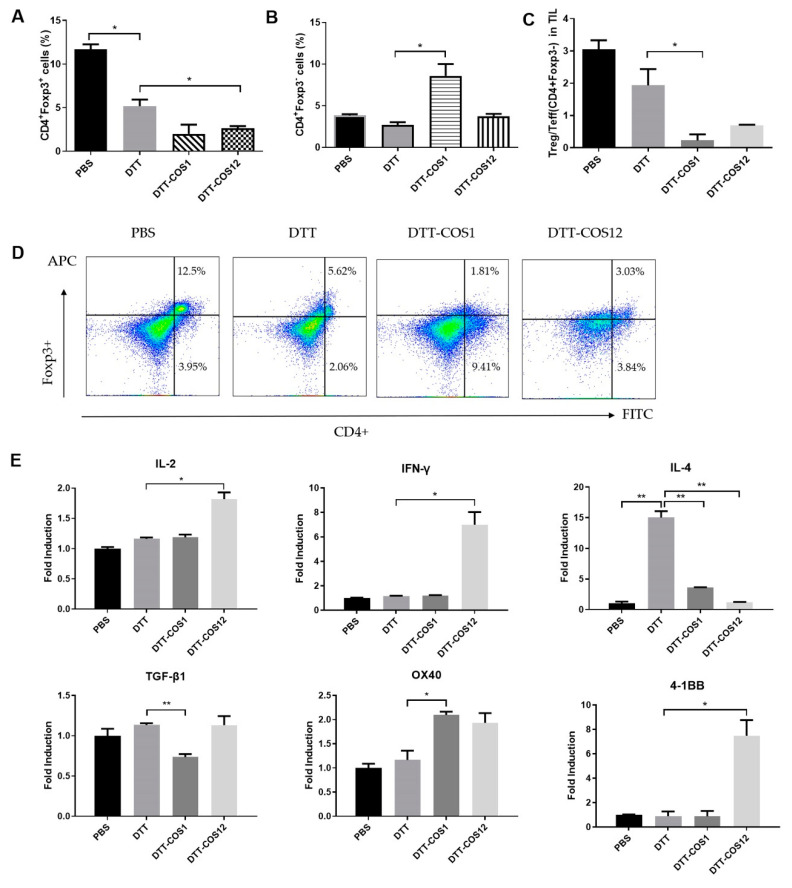
DTT-COS1 and DTT-COS12 decreased the ratio of Treg/Teff and modulated cytokine expression in tumor microenvironment. (A-C) The TILs from DTT, DTT-COS1, DTT-COS12 and PBS groups stained with anti-CD4-FITC and anti-Foxp3-APC. (**A**) The percentages of Treg (CD4+Foxp3+) in TILs. (**B**) The percentages of Teff (CD4+Foxp3-) in TILs. (**C**) The ratio of Treg(CD4+Foxp3+)/Teff(CD4+Foxp3-) in TILs. (**D**) Representative flow cytometry analysis of Treg (CD4+Foxp3+) and Teff (CD4+Foxp3-) in PBS, DTT, DTT-COS1, and DTT-COS12 treatments. (**E**) The mRNA expression of IL-2, IFN-γ, IL-4, TGF-β1, OX40 and 4-1BB in B16F10 tumor tissues isolated from DTT, DTT-COS1, DTT-COS12 and PBS groups. The results are shown as means ± SD and the statistical significance was determined by Student’s T test compared to DTT groups. ** *p* < 0.01; * *p* < 0.05.

Further studies were necessary to explain the surprising antitumor activities of DTT-COS1 and DTT-COS12. Based on the therapeutic model, we analyzed TILs in B16F10. Flow cytometry results showed that treatment with DTT-COS1 (no statistical difference) or DTT-COS12 distinctively reduced the proportion of Treg (CD4+Foxp3+) in TILs (<3.1%), compared to DTT (~5.18%) or PBS (~11.7%) (Figure 4A,C). In contrast, DTT-COS1 treatment showed a marked increase in the percentage of Teff (CD4+Foxp3-) by more than 7% (Figure 4B,C). The proportion of Treg/Teff (CD4+Foxp3-) was visibly reduced in DTT-COS1 (~0.23%) and DTT-COS12 (~0.7%) groups compared with PBS (~3%) or DTT (~1.9%) (Figure 4C). Immunohistochemical staining of tumor tissues showed that DTT-COS12 treatments had a more positive area with anti-CD8 than control (Supplementary Figure S1A,B), and in contrast less positive area with anti-CD4 (Supplementary Figure S1A,C).

We measured the mRNA expression levels of Th1-associated cytokines (IFN-γ, IL-2), Th2-associated cytokines (IL-4), and Treg-associated cytokines (TGF-β1) in TME. IL-2 expression was approximately 1.2-fold and 1.8-fold in the DTT-COS1 or DTT-COS12 treatment compared to PBS, respectively (Figure 4E). Meanwhile, it was also significantly increased in IL-2 expression with DTT-COS12 treatment compared to DTT (Figure 4E). IL-2 expression in DTT was closed to DTT-COS1 (Figure 4E). IFN-γ expression was dramatically increased 6-fold in the DTT-COS12 groups compared to PBS or DTT (Figure 4E). DTT has been reported to relate to humoral immunity in the Th2 polarity pathway [26]. Th2-associated IL-4 expression was greatly increased in DTT only groups (14-fold) and DTT-COS1 treated groups (3.5-fold), while no difference was observed in DTT-COS12 treated groups (Figure 4E). The mRNA expression levels of TGF-β1, a Treg-associated cytokine, decreased by 35% in DTT-COS1 treated groups compared to DTT (Figure 4E). Interestingly, we found that the mRNA expression of OX40 and 4-1BB were different in costimulatory fusion protein treatments. OX40 expression was increased in both DTT-COS1 and DTT-COS12 (no statistical difference) groups, approximately 2.0-fold and 1.8-fold respectively compared with DTT (Figure 4E). While 4-1BB expression was improved in DTT-COS12 treatment only, by 6.5-fold compared with DTT (Figure 4E).

These data indicated that DTT-COS1 could be associate with a reduction of Treg population and TGF-β expression in TME, as well as an increase of CD4+ Teff population in TILs. Meanwhile, DTT-COS12 seems to be more associated with an increase of IFN-γ and IL-2, as well as Treg reduction.

### 3.4. DTT-COS1 and DTT-COS12 Can Generate Endogenous Antibodies Which May Contribute to Protective Effect against Tumors

**Figure 5:**
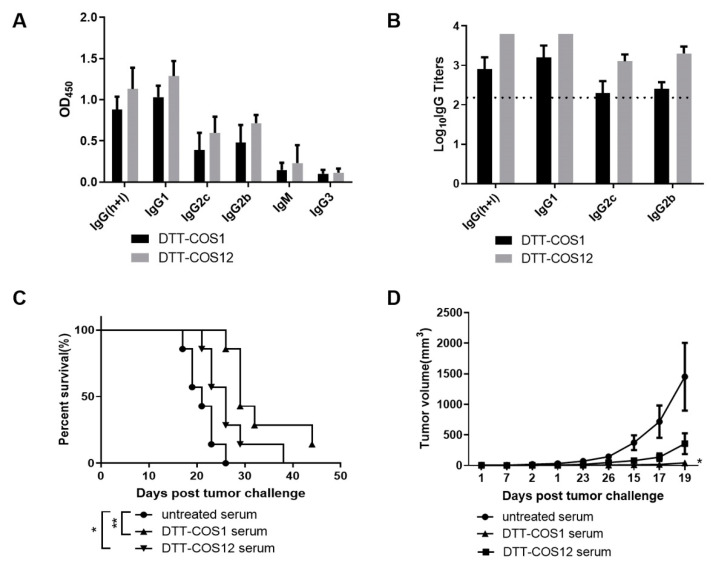
DTT-COS1 and DTT-COS12 generated endogenous antibodies against OX40L and serum transfer may contribute to the protective effect against B16F10. (**A**) The OD_450_ analysis of different anti-OX40L antibody subclasses. The sera were 1:200 diluted. (**B**) The titers of different anti-OX40L antibody subclasses. (**C**,**D**) C57BL/6 mice (*n* = 5–7) were injected i.p. with serum of high-titer anti-OX40L antibody (300 µL/mouse) 6 hours prior to 1 × 10^5^ B16F10 challenge. (**C**) The Kaplan-Meier survival plot of serum transfer. (**D**) Tumor growth curves. Results are presented as means ± SD, and the statistical significance was determined by Log-rank test and Student’s T test. ** *p* < 0.01; * *p* < 0.05.

Antibodies are known to have a variable and direct effect on tumors such as the killing of antibody-bound target cells via antibody-dependent cellular cytotoxicity (ADCC), antibody-dependent phagocytosis (ADP) and though opsonization by antigen presentation and processing of APC [27]. We asked whether pretreatment with proteins can generate endogenous antibodies against OX40L and 4-1BBL and whether such antibodies can contribute to a protective effect against tumors. The DTT-COS1 and DTT-COS12 treatments produced robust antibody responses and high levels of different IgG subclass (IgG1, IgG2b, IgG2c) titers (log10 >2) against OX40L (Figure 5A,B). Similarly, the DTT-COS2 and DTT-COS12 treatments produced different IgG subclass (IgG1, IgG2b, IgG2c) titers (log10 >3) against 4-1BBL (Supplementary Figure S2A,B).

Different IgG subclass reveals different immune pathways [28]. In mice antibody subclass systems, Th1 cells are primarily involved with cellular immunity and associated with switching to IgG2a (IgG2c in C57BL/6) [29,30], while Th2 cells promote switching to IgG1 involved with humoral immunity [31]. Serum containing high antibody titers against anti-OX40L (Figure 5B) was transferred i.p. into C57BL/6 mice (300 μL/mouse) 6 h before the B16F10 tumor challenge. These therapy-induced antibodies were functional, as serum transferred from DTT-COS1-treated and DTT-COS12-treated mice significantly prolonged untreated mice survival and slowed the growth of the tumor under B16F10 challenge (Figure 5C,D). The result indicates that DTT-COS1 and DTT-COS12 generated endogenous antibodies in vivo which may contribute to the protective effect against the B16F10 challenge.

### 3.5. DTT-COS1 and DTT-COS12 as Safe Formulations in Mice

**Figure 6:**
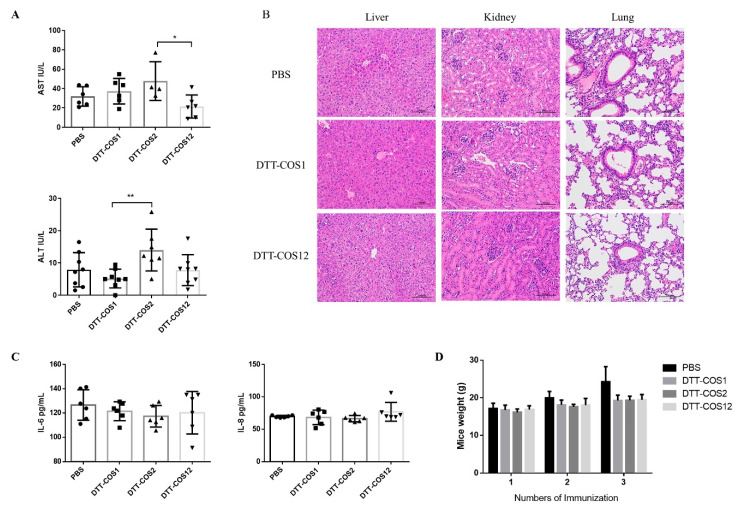
Safety evaluation of co-stimulatory fusion proteins. C57BL/6 (*n* = 6–8/group) were treated with PBS, DTT-COS1, DTT-COS2 and DTT-COS12. (**A**) The serum AST and ALT on the seventh day after third treatment. (**B**) The liver, kidney and lung tissue sections by H&E staining. (**C**) The serum IL-6 and IL-8 levels. (**D**) The body weights of mice in different groups. The results are shown as means ± SD and the statistical significance was determined by Student’s T test compared to PBS groups. ** *p* < 0.01; * *p* < 0.05.

For the safety evaluation of costimulatory fusion proteins, we measured the level of serum ALT and AST activity to reflect damage to hepatocytes [32]. All analysis of serum were from mice treated with DTT-COS1, DTT-COS2, DTT-COS12, or PBS in the presence of aluminum hydroxide Gel adjuvant (300 μg\200 μL; Invitrogen US) and CpG ODN 1826 (30 μg\200 μL; synthesis) on the seventh day after the third treatment. DTT-COS1 and DTT-COS12 had minimal impact on serum ALT or AST levels (Figure 6A), similar to the PBS group. DTT-COS2 significantly increased ALT and AST levels (~14.01 IU/L and ~47.68 IU/L, respectively), compared to DTT-COS1 (~5.186 IU/L) and DTT-COS12 (~21.47 IU/L) (Figure 6A). There were no significant differences between the DTT-COS2 and PBS groups in serum ALT and AST (Figure 6A). The H&E staining showed that the liver, kidney, and lung tissue sections of the mice treated with costimulatory fusion proteins were almost the same as the PBS group (Figure 6B). Serum concentrations of IL-6 and IL-8 were analyzed to show the inflammatory reactions by ELISA [33]. There were no obvious differences between the three proteins and the PBS group in the serum of the two pro-inflammatory cytokines IL-6 and IL-8 (Figure 6C). Increasing the number of treatments, the body weights of mice treated with DTT-COS1, DTT-COS2, or DTT-COS12 did not show any differences with the PBS group (Figure 6D). These results indicate that DTT-COS1 and DTT-COS12 were safe and less toxic to mice.

## 4. Discussion

In this study, we have demonstrated an effective strategy for cancer immunotherapy. Costimulatory fusion proteins, DTT-COS1 and DTT-COS12, protected mice from B16F10 tumor challenge in prophylactic and therapeutic models. Immunomodulation by DTT-COS1 in TME increased the proportion of CD4+ Teff cells and decreased the expression of TGF-β. Meanwhile, immunomodulation by DTT-COS12 in TME reduced the proportion of Treg cells and increased the expression of IL-2 and IFN-γ. The proteins elicited high-titer antibodies against OX40L/4-1BBL without systemic toxicity, which may affect tumor protection.

There have been few reports on the soluble expression of costimulatory ligands in *E. coli* expression systems among previous studies, and one demonstrated the recombinant human 4-1BBL inclusion body as the form of expression [34]. Our study is the first to achieve the soluble expression of OX40L, 4-1BBL, and their tandem forms in *E. coli*. Earlier reports indicated that native costimulatory protein ligands such as native 4-1BBL have no costimulatory activity [35,36], and some studies have fused OX40L or 4-1BBL with different carriers to promote T lymphocytes growth and exhibit potent biologic activity [37,38]. DTT, a transmembrane domain of diphtheria toxin with universal Th epitope, is an effective antigen carrier and scaffold to break immune self-tolerance [24,39,40], also used as a soluble membrane anchor [41,42,43,44]. Here, DTT was used as a carrier protein in our study, which fused with extracellular domains of costimulatory ligands, 4-1BBL, OX40L, and in combination. Our results showed that DTT-COS1 and DTT-COS12 had costimulatory activities on immune cells in vitro, regulating the proportion of CD3+CD4+ and CD3+CD8+ T cells, and DTT-COS12 notably increased the secretion of IFN-γ. DTT-COS12 have one more extracellular domain of 4-1BBL than DTT-COS1. Maybe the function of an additional costimulatory factor, 4-1BBL determined the difference of immune response between naïve and immunized state. As reported in previous reports, costimulatory signals, like 4-1BB/4-1BBL, have a pivotal role in the survival of activated effector and memory CD8+ T cells [45,46,47,48] and are important in establishment and maintenance of the CD8+ T cell recall response to antigens or viruses [48,49]. The stimulation of 4-1BB/4-1BBL preferentially induces Th1 responses by increasing IFN-γ, IL-2, and CD8+ T cell proliferation [16,45]. Therefore, DTT-COS12, a combination of two costimulatory factors, generated a higher immune response in the immunized state and enhanced the production of Th1 associated cytokines, like IFN-γ and IL-2.

Treatment with DTT-COS1 or DTT-COS12 had a long-term protective effect on mice inoculated with B16F10 cells, for up to 60 days, and more than 80% of mice had no tumor growth. This result is consistent with the recently reported preventive efficacy of the OX40 antibody and 4-1BBL fusion proteins [50,51]. Moreover, very few reports have demonstrated the role played by costimulatory proteins alone in the therapeutic model of tumors. To our surprise, after the B16F10 tumor challenge, there was a significantly prolonged survival and delay of tumor growth with DTT-COS1 or DTT-COS12 treatment. Disappointingly, we did not observe any antitumor effects on DTT-COS2 in vivo or in vitro, perhaps with other models. Recently, the combination of the OX40 antibody and CpG has a remarkable antitumor effect in situ [50], but the drawbacks of in situ immunization are the appropriate immune cells infiltration and adequate tumor injection site. A comparison in tumor-challenge trials using CT26 mice models was less effective compared to B16F10 models. Future studies should consider the optimization of doses, tumor models, and the mechanism of research.

Tumor-infiltrating Tregs play immunosuppressive roles in cancer and enhance suppression in the TME [52]. The antitumor mechanism has been reported in OX40/OX40L co-stimulation, preventing the induction of Foxp3+ Tregs from T effector cells [53]. Other studies have shown that OX40/OX40L engagement depleted intratumoral Tregs that correlated with tumor regression [54]. Consistent with previous researches, the function of OX40L showed a decrease of tumor-infiltrating regulatory T cells population in DTT-COS12 treatment. The population of tumor infiltrating CD4+ effector T cells increased distinctly in DTT-COS1 treatment. We also found IFN-γ and IL-2, involved in cellular killing immunity, were sharply increased by DTT-COS12 treatment, while the expression of TGF-β was significantly decreased by DTT-COS1 treatment. As one of the immunosuppressive cytokines, TGF-β inhibits the production and function of effector T cells as well as antigen-presenting dendritic cells (DC) [55,56]. The expression of IL-4, which is associated with humoral immunity [57,58], had no significant change in our experimental groups. Earlier studies have shown that OX40 was highly expressed by intratumoral Tregs [54]. Surprisingly, we have also found an increase in the expression of costimulatory receptor OX40 with DTT-COS1 treatment, and related receptor 4-1BB with DTT-COS12 treatment in TILs, which probably helped the infiltration of effect T cells and tumor resist in TME. We can infer that costimulatory fusion proteins successfully decreased intratumoral Tregs to produce antitumor responses.

There are five antibody subclasses (IgA, IgD, IgE, IgG, and IgM) in mice, which are similar in structure and different in function [59]. We assessed the immune response and polarization of Th1 and Th2 through murine IgG (h+l), IgG1, IgG2b, IgG2c, IgG3, and IgM. Mouse IgG2a/2b (IgG2c in C57BL/6) is considered the same as human IgG1 [30,60], represent cellular immunity (Th1 polarization) [29,30,31]. Mouse IgG1 is considered the same as human IgG4, suggesting humoral immunity (Th2 polarization). IgM is the first immunoglobulin class to be synthesized by the neonate and plays a role in the pathogenesis of some autoimmune diseases [30]. It is reported that the IgM antibody can make a good biologic against cancer owing to its strong avidity, as well as complement fixation property [28]. Mouse IgG3 was demonstrated to be effective against several life-threatening bacterial infections and is the only IgG subclass able to agglutinate the cells when recognizing a surface antigen of red blood corpuscle [28,61]. Based on the results, DTT-COS1, DTT-COS2, and DTT-COS12 treatment produced higher titers of endogenous antibodies subtypes against OX40L and/or 4-1BBL. Serums with a high-titer antibody against OX40L could surprisingly contribute to a protective effect on B16F10 tumors. It has been shown that non-specific IgG substantially accumulates in solid tumors and endogenous IgG was used as a systemic drug delivery to solid tumors and enhance antitumor activity [62].

## 5. Conclusions

In conclusion, we demonstrate the unexpected immunomodulatory characteristics of different costimulatory ligand proteins with no combination of tumor-associated products, which showed potent immune responses against B16F10 tumor challenge in both preventive as well as therapeutic efficacy. This was a unique feature of the recombinant costimulatory molecule as an agonistic OX40/4-1BB antibody did not protect mice against tumor challenge. Moreover, treatments with DTT-COS1 significantly increased the percentage of effective TILs and decreased the expression of immunosuppressive cytokine TGF-β, meanwhile, DTT-COS12 decreased the percentage of regulatory TILs and enhanced the production of Th1-associated cytokines, IL-2, and IFN-γ. DTT-COS1 and DTT-COS12 generated endogenous antibodies that helped antitumor immunity, and without systemic toxicity. Thus, our design of recombinant costimulatory ligand proteins is a promising strategy for preclinical and clinical cancer immunotherapy.

## Supplementary Materials

The following are available online at https://www.mdpi.com/2076-393X/8/2/223/s1, Figure S1: DTT-COS1 and DTT-COS12 increased expression of anti-CD8 TILs and decreased expression of anti-CD4 TILs, Figure S2: DTT-COS2 and DTT-COS12 generated endogenous antibodies against 4-1BBL.
