# Supplementary figures and images for: Recombinant Costimulatory Fusion Proteins as Functional Immunomodulators Enhance Antitumor Activity in Murine B16F10 Melanoma
Source: Vaccines (Basel). 2020 May 14;8(2):223. doi: 10.3390/vaccines8020223 (PMC7349950; doi:10.3390/vaccines8020223)

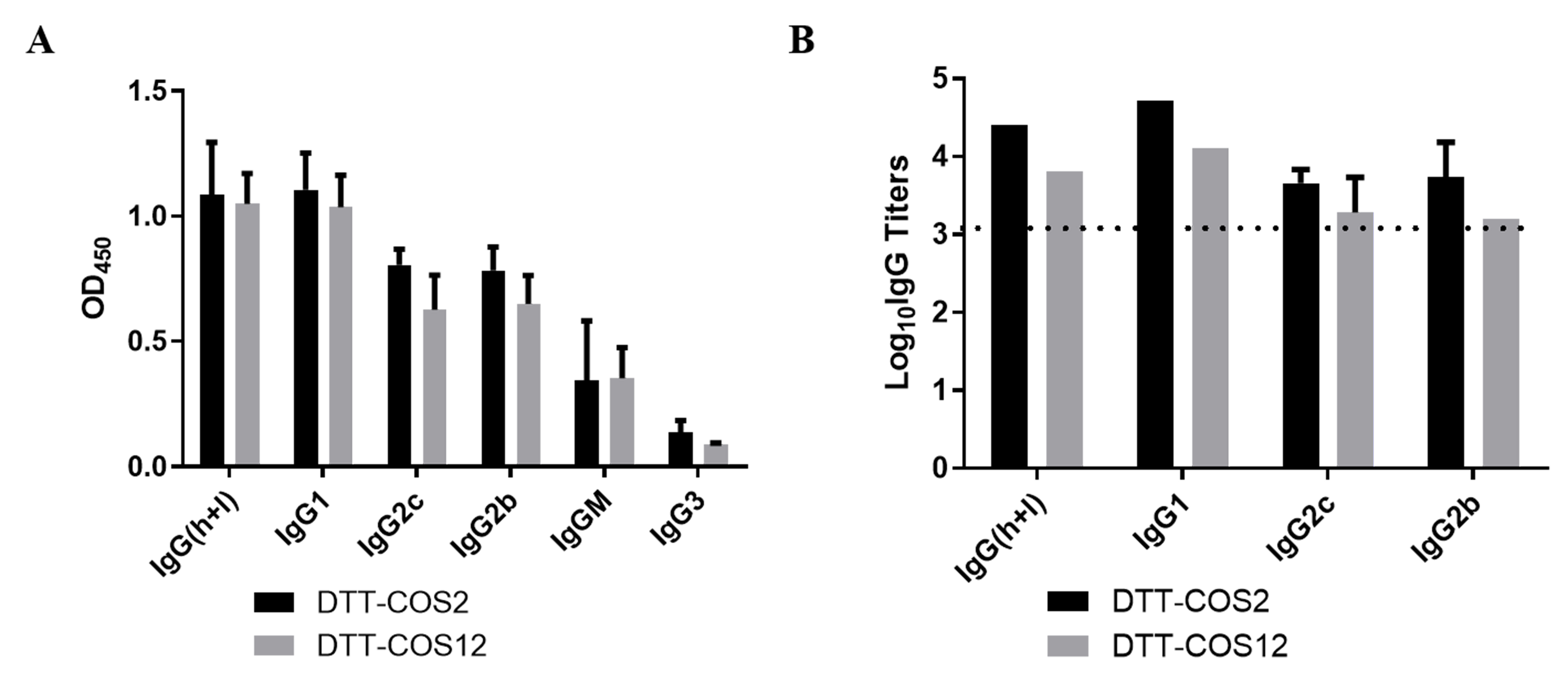

Supplement: Supplementary file 1 [file vaccines-08-00223-s001.zip › Supplementary/Supplementary Fig S2.jpg]
